# Identical fracture patterns in combat vehicle blast injuries due to improvised explosive devices; a case series

**DOI:** 10.1186/1471-227X-12-12

**Published:** 2012-10-10

**Authors:** Joris Commandeur, Robert Jan Derksen, Damian MacDonald, Roelf Breederveld

**Affiliations:** 1 Department of surgery/ traumatology, VU medical center, De Boelelaan 1117, 1081, HV, Amsterdam, The Netherlands; 2 Department of surgery/ traumatology, Red Cross Hospital Beverwijk, Beverwijk, The Netherlands; 3 Department of surgery/ traumatology, 1 Canadian Field Hospital Ottawa Detachment, Canadian Forces Health Service, Ottawa, Canada

**Keywords:** Improvised explosive devices, Identical injuries, Blast injury

## Abstract

**Background:**

In November 2008, a surgical team from the Red Cross Hospital Beverwijk, the Netherlands, was deployed in Afghanistan for three months to attend in the army hospital of Kandahar.

During their stay, four incidents of armored personnel carriers encountering an improvised explosive device were assessed. In each incident, two soldiers were involved, whose injuries were strikingly similar.

**Case presentation:**

The described cases comprise paired thoracic vertebral fractures, radial neck fractures, calcaneal fractures and talar fractures. Moreover, the different types of blast injury are mentioned and related to the injuries described in our series. Acknowledging the different blast mechanisms is important for understanding possible injury patterns.

**Conclusion:**

From this case series, as well as the existing literature on injury patterns caused by blast injuries, it seems appropriate to pay extra attention to bodily areas that were injured in other occupants of the same vehicle. Obviously, the additional surveillance for specific injuries should be complementary to the regular trauma work-up (e.g., ATLS).

## Background

In November 2008, a surgical team from The Red Cross Hospital Beverwijk, the Netherlands, went to Afghanistan to attend in the army hospital of Kandahar Air Field (KAF). During the three-month stay, several armored personnel carriers, type MRAP, encountered improvised explosive devices (IEDs). IEDs are homemade explosives that are often used by insurgents and terrorists in the Middle East. In Iraq, in 2005, 10,000 attacks were reported. From June 2003 to January 2008, IEDs caused over 1,500 fatalities. IEDs are similar to mines and are often activated by the victim himself. Often, IEDs incorporate metal fragments and/or animal fecal excrements [1-4]. IEDs contributed to the majority of injuries in casualties in the British Military Field Hospital, Shaibah, Iraq in 2006 [5].

Upon the victims’ arrival in the hospital, after triage, resuscitation and stabilization, it became clear that the occupants in each vehicle had sustained strikingly similar injuries. In this report we will describe the four cases and the trauma mechanisms.

To comprehend the trauma mechanisms, it is important to be well aware of the different types of blast trauma and their impact.

Blast injuries can be classified into four types. Primary blast injuries (explosive forces) are those caused by the direct effect of overpressure on a person. Secondary blast injuries are injuries caused by the effect of projectile fragments incorporated in the bomb, like nails, rocks or scrap metal. Tertiary blast injuries are caused by the effects from the blast wind, resulting in physical displacement. Also in this group are injuries resulting from collapsing buildings. Most fractures, blunt trauma and tissue contusions are tertiary blast effects [1,2,6]. A variety of injuries are classified in the group of quaternary blast injuries, including burns, psychological trauma, toxic inhalation and exposure to radiation [2,6]. The cases described below are classified in the tertiary injury group.

Furthermore the magnitude of the effects of an explosion on a person is dependent on several factors. Most important is the magnitude of the explosion, the medium through which the pressure wave passes, the distance of a person to the epicenter and, lastly, the environment of the incident (i.e., open air or enclosed space) [2,7,8].

The aim of the article is to establish whether useful adjuncts in the assessment of blast injury patients can be put forward following the assessment of four paired cases of blast injury.

## Case presentation

### Case pair A

**Figure 1:**
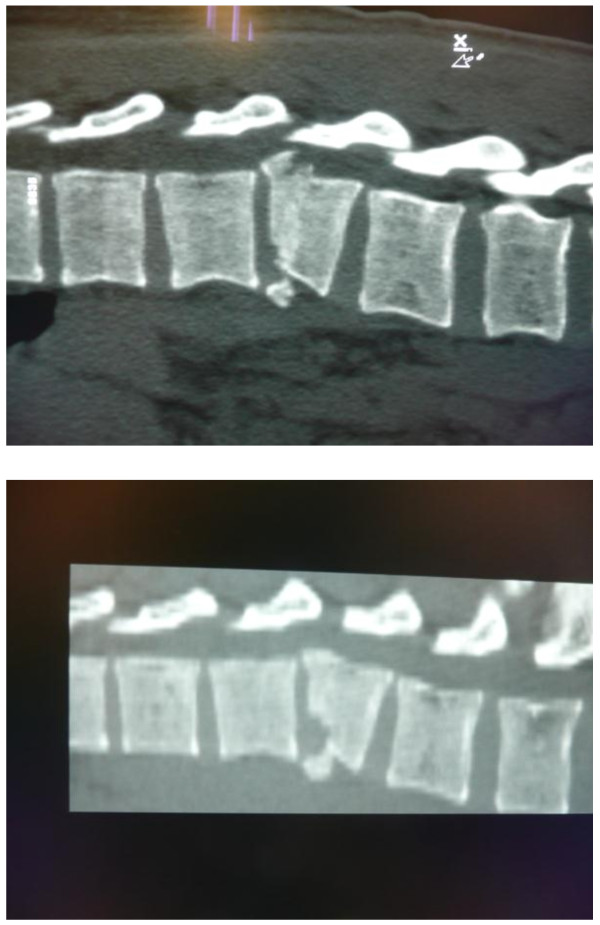
**Case pair A, two sagittal reconstructions of CT-scans of two separate thoracic vertebral columns of two passengers of an armored personnel carrier that hit an improvised explosive device (IED).** Both showed identical, unstable burst-split fractures of the 9^th^ thoracic vertebra.

An armored vehicle was hit by an IED strike. The two soldiers sitting on the front seat of the vehicle were hemodynamically and respiratory stable. Both men complained of back pain and on physical examination palpation of the lower thoracic vertebrae elicited pain. No abnormal neurologic signs were found on examination. A CT scan revealed unstable fractures, Magerl/AO spine fracture classification type 3.2, burst-split, of the anterior and intermediate columns of the 9th thoracic vertebra in both patients (Figure 1). Presumably, a large blast force from beneath pushed their bodies up in their belts, resulting in this type of burst-split fracture. Although lumbar fractures are seen more frequently in sub-vehicle blast injuries, both fractures concerned Th 9 [9,10]. The Abbreviated Injury Score (AIS) was 3 [11].

In Afghanistan, both patients were treated conservatively. Within 48 hours they were transported to Landstuhl, Germany, for additional treatment.

### Case pair B

**Figure 2:**
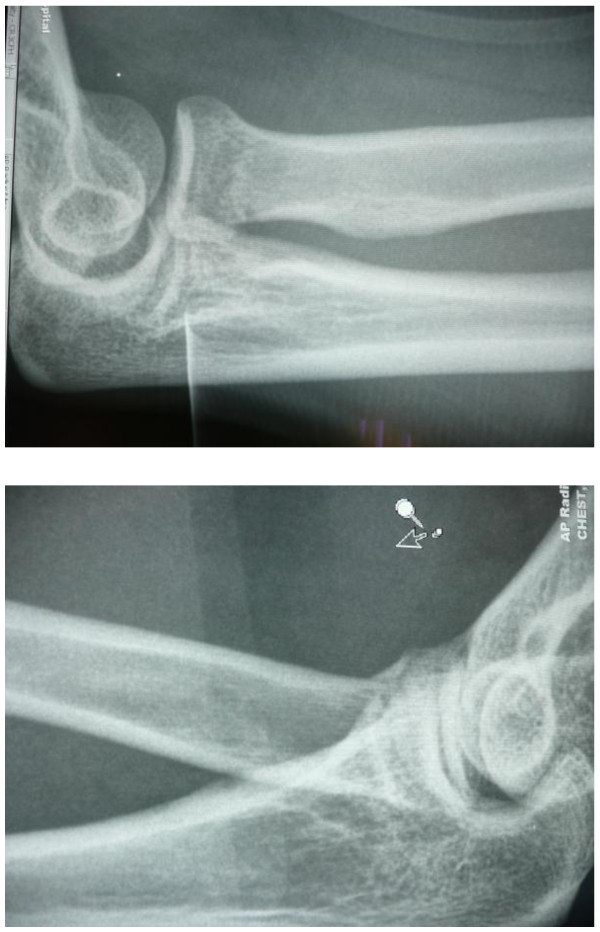
**Case pair B**, **two radiographs of the elbow of two injured passengers of the same armored personnel carrier after having hit an improvised explosive device (IED), showing contralateral fractures of the radial neck.**

Two soldiers, both board gunners, were sitting behind their weapons (attached to the vehicle) on the right and left sides of the truck, holding their weapon in the same way, both hands positioned on a grip. Axial forces injured both soldiers after their truck hit an IED. ATLS work-up did not reveal any airway, respiratory or circulatory instability. In addition to multiple open wounds of the face and hands, they complained of elbow pain. In both cases, X-rays revealed the same radial neck fracture, AO 21-A2.2, slightly displaced (Figure 2). The fact that the soldiers were holding weapons, which were attached to the vehicle contributed to this kind of injury, otherwise when soldiers were thrown around in the vehicle, one would expect other injuries. The AIS was 2 [11].

Both soldiers were treated conservatively.

### Case pair C

**Figure 3:**
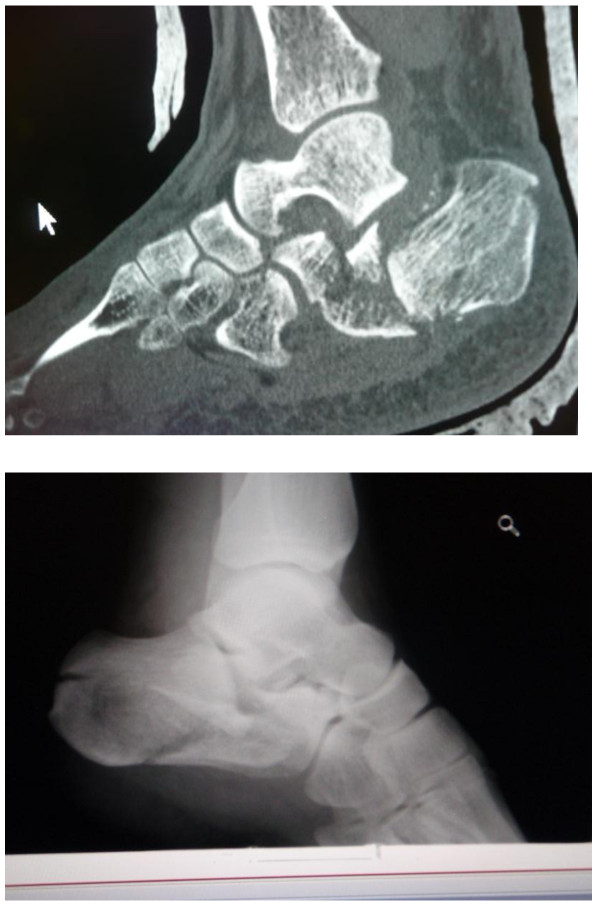
Case pair C, sagittal reconstruction of a CT-scan and a radiograph of the hind foot, showing complex fractures of the calcaneus of two passengers of the same armored personnel carrier after blast injury (improvised explosive device).

In this vehicle, also after an IED attack, there was a significant displacement of the base of the truck. Both soldiers sustained a direct blow from beneath directly to the calcaneus. Again, primary assessment did not reveal vital injuries, and the patients were hemodynamically and respiratory stable. On secondary survey, both men complained of heel pain and on physical examination, swelling and discoloration surrounding the heel was seen. Pain was elicited by axial compression. Radiography showed comminuted, displaced fractures of the calcaneus in both patients, type Sanders 4 (Figure 3). Unexpectedly, they did not sustain other injuries, which would have been expected according to a previous report of Ramasamy et. al. concerning ‘deck-slap’ injuries [12]. The AIS was 3 [11].

Both soldiers were transported to the US, where scopic surgery was performed.

### Case pair D

**Figure 4:**
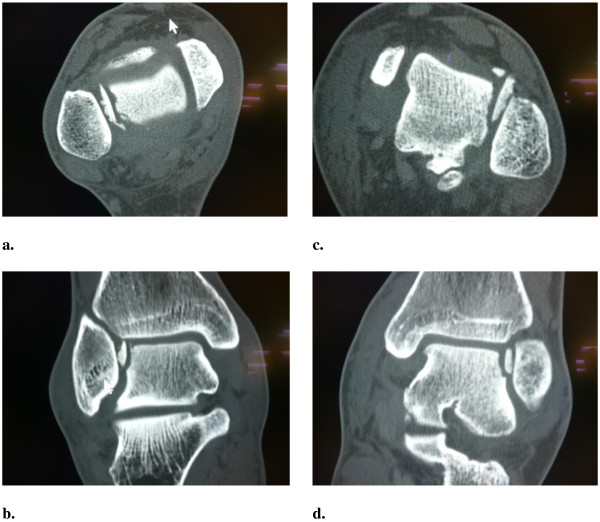
**Case pair D, paired CT-scan images (a and c transverse plane, b and d coronal reconstructions) of the talus.** Images **a** and **b** from the left board gunner, **c** and **d** from the board gunner on the right. Both occupants sustained an inverted osteochondral chip fracture of the lateral talus dome.

Two soldiers, both board gunners were standing behind their weapons on the left and right side of the truck. During an IED strike, the bottom of their vehicle struck their lower legs by a direct blow, caused by the vertical forces of the explosion just below their vehicle. After initial ATLS assessment, both patients were respiratory and hemodynamically stable. During the regular trauma work-up, both patients, although protected by heavy army boots, complained of pain in the ankle joint of the weight bearing leg. Radiographs of the ankles showed an irregular surface of the talus. A CT-scan, showed an unusual flake fracture of the lateral talar wall with 180-degree rotation of the fragments in both patients, type Müller AO/OTA C1 (Figure 4). The AIS was 3 [11]. Both soldiers were operated in the US.

## Discussion

As described in the background, the distance to the blast center plays an eminent role in the severity and type of injury [6]. In the cases described above, the occupants were approximately at the same distance from the blast center, which could partially explain why the impact of the explosion was similar. Furthermore, in each case, both occupants sustained injuries caused by the same blast injury pattern, namely the tertiary type.

The blast wave, coming from an IED, interacts with the vehicles by coupling energy from the blast field into the vehicle [13]. It is clear that the entire vehicle is being exposed to the same amount of energy. This case series shows that strikingly similar and unusual injuries could occur to patients seated in the same vehicle, hit by an explosion.

In all cases, the involved vehicles were MRAPs (Mine Resistant Ambush Protected), their weight is approximately 20,000 kilogram, equipped with armor and glass protection and specialized v-shaped hull design, which especially is developed to protect vehicles against IEDs.

All patients were male US soldiers. After performing damage control surgery in the army hospital in Kandahar, injured soldiers are transported to their home country or to the Landstuhl Regional Medical Center in Germany, a military hospital operated by the United States Army and the Department of Defence.

Based on the described cases, since injuries were found that were unexpected and paired, a thorough secondary and tertiairy survey with special attention for injured bodily areas of the codriver is essential. To improve the trauma work-up, one should be well aware of the trauma mechanism and its consequences.

A literature search on identical orthopedic injuries after blast trauma yielded one report: in 2002 in Karachi, Pakistan, 12 survivors of a suicide bombing of a bus were brought to a private tertiary university hospital. Of these twelve survivors, all had lower limb fractures, including eleven who had fractures of the foot and ankle region and seven who suffered bilateral calcaneal fractures. Remarkable was that five of them had a Gustilo-Anderson grade III A calcaneal fracture (widespread damage of soft tissue, muscle, skin and neurovascular structures, but adequate soft-tissue coverage of the fractured bone [14]). It is important to know that the suicidal motorist hit the bus from the side and below, which implies that the blast wave came from a lower level than the victims [15].

## Conclusion

From the striking similarities in the paired trauma cases of blast injuries, we conclude that special attention in the secondary and tertiary survey should be focused on bodily areas that are injured in the co-driver.

## Consent

I, Roelf Breederveld declare that all soldiers agreed with the anonimized publication of the radiographs and CT-scans in a report or elsewhere. A verbal consent was obtained. Due to rush, high turn-over in the hospital it was not possible to obtain written consent of the soldiers. Roelf Breederveld.

## Competing interests

The authors declare that they have no competing interests.

## Authors’ contributions

JC and RJD wrote the case report. DMcD made some major changes after reviewing the first version. RB supervised the writing of this paper and made some major changes after reviewing the versions. All authors read and approved the final manuscript.

## Pre-publication history

The pre-publication history for this paper can be accessed here:

http://www.biomedcentral.com/1471-227X/12/12/prepub
